# Prediction of traffic accidents trend with learning methods: a case study for Batman, Turkey

**DOI:** 10.1038/s41598-025-11835-9

**Published:** 2025-07-22

**Authors:** Enes Bakiş, Mehmet Ali Erçetin, Emrullah Acar, İslam Gökalp, Musa Yılmaz

**Affiliations:** 1 Research Assistance, Faculty of Engineering, Department of Electrical and Electronics Engineering, Piri Reis University, Tuzla, 34940 Istanbul, Turkey; 2 Kozluk Vocational School, Department of Construction, Batman University, Kozluk, 72400 Batman, Turkey; 3 Department of Electrical and Electronics Engineering, Faculty of Engineering and Architecture, Batman University, City Center, 72000 Batman, Turkey; 4 Department of Civil Engineering, Faculty of Engineering and Architecture, Batman University, City Center, 72000 Batman, Turkey; 5 Center for Environmental Research and Technology, Bourns College of Engineering, University of California at Riverside, Riverside, CA 92521 USA

**Keywords:** Traffic accident, Prediction, Learning methods, Hybrid models, Civil engineering, Electrical and electronic engineering

## Abstract

Assessing the trend of fatalities in recent years and forecasting road accidents enables society to make appropriate planning for prevention and control. This study analyses the road traffic accident data between the years 2013 and 2022 obtained for the province of Batman in Turkey, where it has not been considered before. The scope of the data analysed includes the fatalities and injuries of drivers, passengers and pedestrians. The road accident forecast for the next ten years up to 2032 is the focus of this study and numerous analyses using learning methods such as State Space Models (SSM), Artificial Neural Networks (ANN), Autoregressive Integrated Moving Average (ARIMA) and hybrid models (CNN + LSTM and Attention + GRU) have been performed on the available data. The predictions made with the above models give results with acceptable accuracy. However, they give different results depending on the parameters used. The models created with the data studied show that the number of road accidents and the related deaths and injuries will continue to increase over the next 10 years, starting in 2022. If the causes of road accidents are not eliminated and the situation remains stable as it is in 2022, the number of accidents, deaths and injuries is expected to double by 2032.

## Introduction

Traffic accidents are a serious problem that endangers both property and life safety^1,2^. According to the report published by the World Health Organization (WHO), approximately 1.19 million people died in traffic accidents in 2021. Compared to 2010, when there were 1.25 million deaths, there was a 5% decrease in death in 2021. Most United Nations member states have achieved a reduction in road traffic fatalities between 2010 and 2021. Although the number of motor vehicles has more than doubled globally, road networks have expanded significantly and the population has increased by nearly one billion, the overall decline in deaths has been limited. This shows that efforts to improve road safety are effective. The United Nations Decade Action Plan on Road Safety (2021–2030) reveals that the interventions needed to achieve the goal of halving deaths by 2030 are still insufficient^3^. At the same time, approximately 20 to 50 million people suffer non-fatal injuries as a result of traffic accidents each year^4^. According to Turkish Statistical Institute (TSI) data, approximately 1.23 million accidents occurred in Turkey in 2022. In these accidents, 5229 people lost their lives and approximately 300 thousand people were injured. In the period from 2013 to 2022, there is an increase of approximately 22% in the number of fatal and injury accidents^5^. Moreover, traffic accidents affect the gross national product of countries by approximately 3%^6^.

The occurrence of traffic accidents depends on numerous various factors^7–9^. Some of these factors are based on driver and pedestrian characteristics. Some examples include driver skills, experience, alcohol and/or drug use, obeying the rules, and speeding tendency^10,11^. Others consist of road physical standards and environmental conditions. These include road volume, geometry (slope, horizontal curve, lane width, shoulder width), type (divided, undivided), conditions (pavement type and potential deterioration of the pavement surface), weather conditions (wind, ice, snow, fog, rain, etc.)^12–14^. All these factors, individually or a couple of them together, may cause fatal traffic accidents. In order to prevent or reduce the occurrence of future accidents, it is very important to develop policies and take the necessary actions. If a forward-looking forecast or prediction is made based on available data, the seriousness of the current situation can be revealed and the necessary measures can be taken immediately^13^.

Accident prediction models are important in terms of providing a future perspective. The models offer valuable guidance to engineers and planners in the absence of real-time driving data, despite the wide range of causes of traffic accidents^15^. These models analyze accidents that occur over a specific period and attempt to build statistical models by relating them to various risk factors^16^. At this point, identifying potential measures for future scenarios requires developing a comprehensive strategy that includes factors such as driver education, strengthening traffic rules, and improving road infrastructure^17^. These measures can help solve current problems and minimize future accidents by improving traffic safety^18–20^.

Accident data can include different accident characteristics that comprise a large database^21^. Various analytical methods are used in the literature to analyze this database^14^. One of these methods is the data mining technique^22^. Data mining uses a variety of tools to analyze accident data, including database technology^23^, statistics^24^, machine learning^25^, high-performance information processing^26^, pattern recognition^27^, neural networks^28^, data visualization^29^, information retrieval^30^, image and signal processing^31^, and spatial data analysis^32–35^. These models use statistical modeling techniques to identify correlations between variables that are difficult to establish directly^36,37^. In recent years, machine-learning theory has been widely used in text, image and voice recognition^38,39^.

In the prediction of traffic accidents, machine-learning methods are utilized based on the ability of machine learning methods to process multidimensional data, flexible application, coding capability and strong prediction capabilities^40,41^. In these models, researchers try to predict the probability of an accident occurring with the help of a defined set of conditions and variables, taking into account the road accident area^42,43^. Among these methods, the most widely used are: Naive Bayes (NB)^44^, k-nearest neighbor (K-NN)^45^, logical regression (LR)^46^, deep residual neural networks (DRRNNs)^47–49^, random forest (RF)^50^, decision tree (DT)^51^, support vector machine (SVM)^52^, multivariate negative binomial (MVNB)^53^, ANN^54^, deep learning^55^, state space model (SSM)^56^, ARIMA^57^ and long short term memory (LSTM)^58^ algorithms.

## Literature review

In this section, numerous available studies presented to make clear prediction models that used for future perspective of traffic accidents based on available databases.

Gatarić et al. (2023) used limited data sets to predict traffic accidents on main roads in Serbia and Bosnia and Herzegovina using ANN methods. Two different ANN models were used to predict traffic accidents causing fatalities, injuries and property losses by considering factors such as road length, terrain type, road width, annual average daily traffic (AADT) and speed limits. The prediction accuracy of traffic accidents in the models were determined close to each other as 0.969 and 0.990^10^. Yeole et al. (2022) used accident data from 2014 to 2019 on Indian highways to predict accidents using ANN model and multiple linear regression (MLR) techniques. The inputs for building the ANN model are weather conditions, vehicle load conditions, number of lanes and accident periods, traffic signs, speed humps and road intersections. The results showed that the accuracy of the prediction model using multiple regression was 88%, while that of the ANN was 93%^59^. Similarly, Alqatawna et al. (2021) used ANN and multivariate regression methods to analyze and predict traffic accidents in regions of Spain with high accident rates. It is revealed that the accident prediction made with the ANN model that was developed with 2014–2017 traffic accident data based on factors such as road segments, years, road length, AADT, horizontal curve radius and other variables are very close to the actual accident data^60^. Alkheder et al. (2017) used data from 5973 traffic accidents in Abu Dhabi between 2008 and 2013 to predict accident injury severity using a developed ANN model. To achieve this, four injury severity classes (fatal, severe injury, moderate injury, minor injury) were identified with 16 variables in the accident records. They reported that the accident severity was predicted with an accuracy of 74.6% using the developed ANN model. An ordered probit model was used to verify the performance of the ANN model, but it was reported that this model was able to predict accident severity with 59.5% accuracy^15^. García et al. (2018) compared accident injury severity with ANN and BN models using data on traffic accidents in Switzerland between 2009 and 2012. The MAPE analysis results for the ANN and BN models developed within the scope of the study revealed an accuracy of 22.4% and 21.8% for minor injury, 27.0% and 27.5% for severe injury, and 30.0% and 51.8% for fatal accident, respectively^61^.

Al-Masaeid and Khaled (2023) used regression, ARIMA and ANN models to predict future traffic accidents, injuries and fatalities in Jordan using traffic accident data from 1995 to 2020. According to the accuracy of the models, ANN models gave the best results, then ARIMA and regression models. The accuracy obtained for the developed ANN models were 0,984, 0,912 and 0.96 for traffic accident, injury and death models respectively^62^. Dutta et al. (2020) used the ARIMA model to predict traffic accident fatalities in India using data covering the period between 1967 and 2015. The ARIMA (0, 2, 1) model used in the analysis implies that there will be a significant increase in the annual total number of deaths due to traffic accidents in India^63^. Getahun (2021) aimed to predict the trend of traffic accidents in the Amhara region of Ethiopia covering the period between September 2013 and May 2017. According to the analysis using ARIMA models, it was concluded that the number of injury, fatal and total traffic accidents will continue to increase over the next 48 months^64^.

Qian et al. (2020) compared the MAPE values of the Elman recurrent neural network (ERNN) and the seasonal autoregressive integrated moving average (SARIMA) models to evaluate road traffic accidents in China and make short-term predictions. The study found that both models performed similarly and effectively in predicting traffic accidents, in which MAPE value is 4.83 for ERNN and 5.04 for SARIMA model^65^. In a similar way, Deretić et al. (2022) analyzed traffic accidents in Belgrade, Serbia from 2016 to 2019 using the SARIMA model. The study demonstrated acceptable performance with a MAPE value of 5.22^66^. Husin et al. (2021) used ARIMA and SSM models to predict monthly traffic accident cases in Malaysia and to determine future trends. The study analyzed the monthly accident data set from January 2001 to December 2019. The analyses show that SSM is the most appropriate model. In addition, projections for 10 years between 2020 and 2030 reveal a trend of a steady increase every year^67^. Junus et al. (2015) used time series regression and SSM-based structural time series methods to model traffic accidents that occurred between 2001 and 2013 in Panang, Malaysia. The result showed that SSM-structural time series gives better results in traffic accident prediction^68^. Dong et al. (2019) proposed an innovative method to predict traffic accidents in their study. This method analyzed traffic accidents by using MVNB, SVM and SSM-SVM in modeling using a five-year data set in the state of Tennessee, USA. According to the compared analysis results, the MAPE values were 14.147%, 11.840%, and 3.522%, respectively. In this case, the SSM-SVM model showed better predictive accuracy compared to the other models^36^. Dutta et al. (2021), analyzed traffic accident data in India from 1967 to 2015 using the exponential smoothing state space model. The research predicted the number of traffic accident fatalities for the next 10 years using the proposed model and found an increased trend^69^. Antoniou and Yannis (2013), conducted a comprehensive analysis of traffic accident data in Greece from 1960 to 2011 using the SSM model. The study compares the prediction results with the actual data from 2009 to 2011 and demonstrates that the model performs well, even under unusual circumstances such as the severe financial crisis in Greece. Additionally, the study includes prediction results up to 2020^70^.

Jiang et al. (2020) developed an LSTM-based accident detection model that considers different time intervals of traffic data from the performance measurement system for highways in California, USA. The developed model has a higher accuracy rate of 70.43% compared to other machine learning methods such as LR, RF, K-NN, SVM, NB and ANN^71^. Li et al. (2020) analyzed traffic accident data at road intersections in Florida, USA between September 2017 and September 2018 using various models, including LSTM-CNN, LSTM, CNN, XGBoost, and Bayesian Logistic Regression. The study found that the LSTM-CNN model outperformed the other models in terms of area under the curve (AUC), sensitivity, and false alarm rate^58^. Sameen and Pradhan (2017) developed a RNN model with a LSTM layer to predict injury severity in traffic accidents in Malaysia. The study compared the performance of this model with that of multilayer perceptron and Bayesian logistic regression models and showed that the proposed RNN model outperformed the other models with an accuracy of 71.77%^72^. Looking at the studies in the literature on road accident prediction, it can be seen that ANN, ARIMA, and SSM models are widely employed due to their capability in handling complex data structures and providing reliable forecasts. Recently, several researchers have demonstrated significant methodological improvements. For instance, Wen et al. (2021) highlighted the superior accuracy of ANN models in predicting accident severity under diverse traffic volumes and weather conditions^73^. Likewise, Katambire et al. (2023) compared ARIMA models in various urban contexts, identifying key predictive factors such as traffic density and seasonal variations^74^. Wang et al. (2021) introduced advanced SSM specifically tailored for traffic safety data, improving prediction robustness by effectively managing time-dependent variables^75^. These recent contributions underline the importance of continuously refining prediction methods to enhance their practical applicability.

## Motivation and scope

It was expressed that traffic accidents are one of the most important problems of the society causing loss of lives and property. The biggest shareholder in the occurrence of traffic accidents is the human being, which includes pedestrians, passengers and drivers. Studies show that 90–95% of traffic accidents are caused by human errors. For this reason, it is aimed to reveal the damages at past, present and future caused by traffic accidents in terms of deaths and injuries, and the scope of the study is limited to this. It is an important issue to put forward the projection in the future period in order to accelerate the efforts to reduce traffic accidents and the severity of loss of life and property because of these accidents. As far as we know, there is no such study for the province of Batman and this motivated us to carry out this study. To predict the future (2032) projection of traffic accident occurring in Batman, Turkey, the available 10 years (2013–2022) traffic accident data were used. The analyses were done with numerous models including SSM, ANN and ARIMA. It is hoped that this study will raise awareness by reporting the situation to be revealed for the future. Moreover, the study will contribute to the improvement of the current situation with the actions to be taken by the authorized units to which the study will be shared.

## Data collection and methodology

### Data collection

**Fig. 1:**
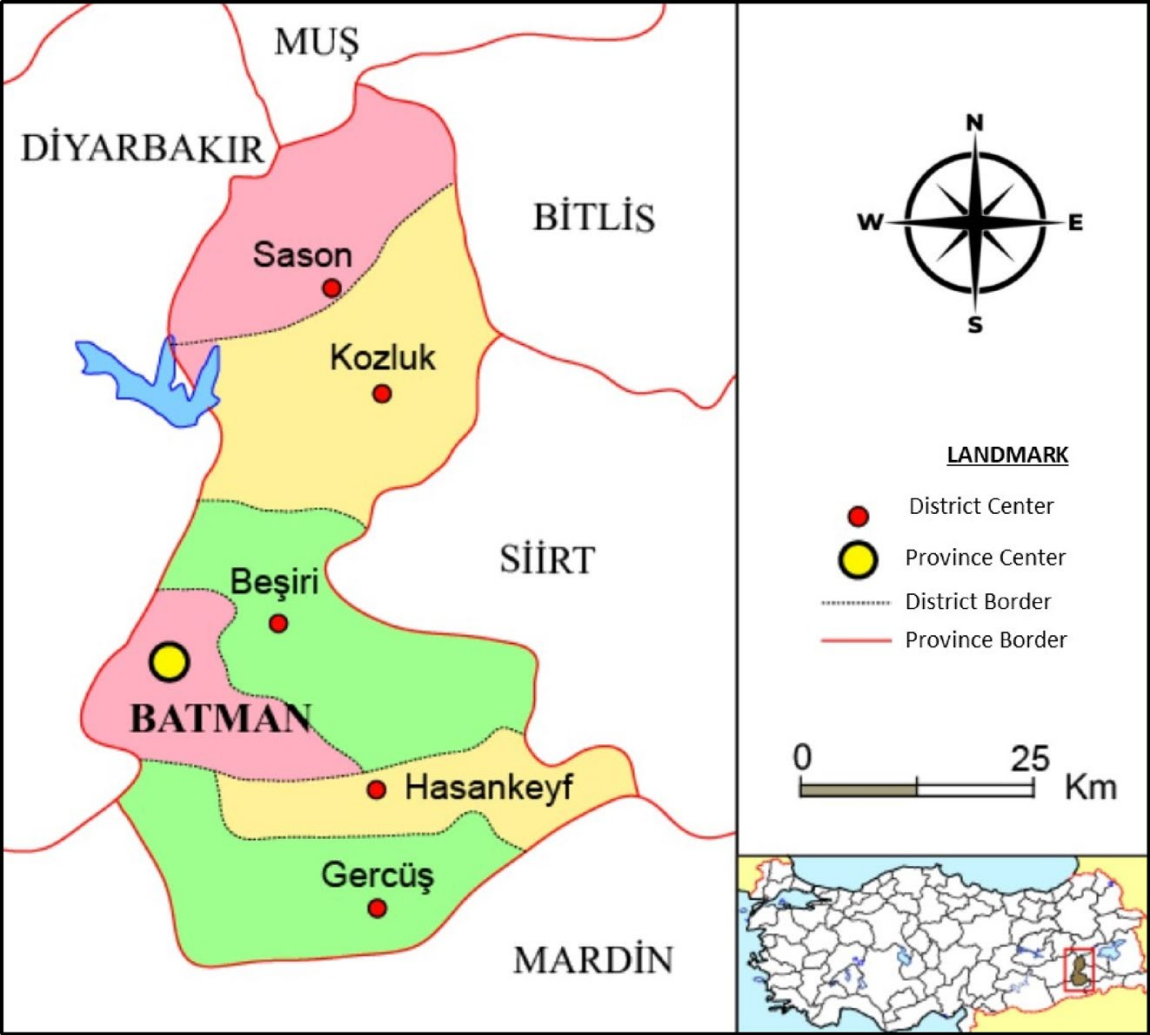
Map of Batman, Turkey.

The data of the present study are secondary and collected from the Ministry of Interior of the Republic of Turkey, General Directorate of Security for Batman, Turkey (Fig. 1).

Researchers collected information about annual basis deaths and inquiries due to road accidents in Batman covering the period from 2013 to 2022. The database includes both the deaths and injuries based on annual reports with the reasons. However, the studied parameters are including driver fatalities and injured driver, passenger, and pedestrian with total accidents. Table 1 showed the traffic accident data and parameters taken into consideration in this study.

**Table 1:** Traffic accident data.

Year	Driver Fatalities	Passenger Fatalities	Pedestrian Fatalities	Injured Driver	Injured Passenger	Injured Pedestrian	Total Fatalities	Total Injuring	Total Accident
2013	4	2	2	373	399	133	8	905	545
2014	5	2	5	846	787	238	12	1727	1018
2015	5	3	6	1222	1240	329	14	2670	1539
2016	7	5	9	1636	1700	389	21	3648	2062
2017	8	7	11	2069	2216	481	26	4783	2647
2018	10	7	12	2492	2725	754	29	5856	3254
2019	11	10	15	2916	3189	913	36	6970	3926
2020	11	13	15	3288	3859	1032	39	7788	4476
2021	11	13	18	3721	4227	1178	42	8771	5119
2022	13	19	19	4177	4608	1328	51	9815	5776

As seen in Table 1 that the accident increased from 545 to 5776 and as a result of them Total Fatalities from 8 to 51, whereas total injuries from 905 to 9815. The trend in traffic accidents over the years is totally upward.

### Methodology

In the light of available studies, highlights the global issue of traffic accidents and their potential threat to life and property. It presents a study that utilizes machine learning modeling methods, including SSM, ANN, and ARIMA and hybrid models (CNN + LSTM and Attention + GRU) to analyze and predict traffic accidents. The models are used to identify statistical relationships between various risk factors and the causes of traffic accidents. In this section these methods are introduced.

#### State space models (SSMs)

SSMs are probabilistic graphical models that describe a system’s dynamics over time. They have a wide range of applications across numerous fields, including time series analysis, econometrics, control systems, and machine learning^76^.

SSMs provide a flexible framework for modelling complex processes and offer several key advantages. They consist of two main elements: the state equation, which explains the fundamental dynamic process, and the observation equation, which links observed data to the hidden states. This hierarchical structure makes SSMs suitable for modelling complex dependencies within systems. SSMs are mathematical models that include latent variables representing the unobserved state of a system. These latent states contain data that is not directly visible in the collected information. SSMs accurately compute and deduce hidden states from data, and facilitate parameter estimation, including the dynamics of state transition and observation noise. Model parameters can be estimated from data using techniques such as maximum likelihood estimation (MLE) or Bayesian inference. SSMs can be used for state prediction, making them a valuable tool for decision-making. They allow for the projection of future states of a system and can be expressed within a Bayesian framework, enabling probabilistic modelling, quantification of uncertainty, and sequential updating of beliefs as new data is obtained. The mathematical model of a State Space Model is generally represented as follows:

State equation:

1 $$\:x\left(t\right)\:=\:F\left(x\right(t-1),\:u(t),\:w(t\left)\right)$$

Observation equation:

2 $$\:y\left(t\right)\:=\:H\left(x\right(t),\:v(t\left)\right)$$

where,

$\:x\left(t\right)=\:$ represents the hidden state at time t

$\:F$ = the state transition function

$\:u\left(t\right)=$ the control input

$\:w\left(t\right)=$ the process noise

$\:y\left(t\right)=$ the observed data at time t

$\:H=$ the observation function

$\:v\left(t\right)=$ the observation noise

Initial state:

The initial state, $\:x\left(0\right)$, is often given a prior distribution

Noise models:

The process noise, $\:w\left(t\right)$, and observation noise, $\:v\left(t\right)$, are typically modeled as random variables with known probability distributions

Parameter estimation:

Model parameters, such as the transition matrix in the state equation and the observation matrix in the observation equation, are estimated from data.

SSMs are a versatile tool with a broad range of applications, from time series prediction and econometric modelling to control systems and beyond. This framework provides a principled basis for comprehending and modelling complex systems that have hidden states.

#### Artificial neural networks (ANNs)

ANNs are machine learning models inspired by biological neural systems. They are efficient in processing data and making decisions, and have various applications in fields such as pattern recognition, prediction, and classification^77^. The input layer introduces the input data, with each input reflected in a neuron. The weight matrix consists of weights that express the connections between inputs and outputs, which are updated during the model’s learning process. The final neuron outputs are computed by subjecting the inputs to an activation function, such as ReLU, sigmoid, or tanh. The ultimate predictions and classifications are produced by neurons in the final layer. An artificial neural network is a mathematical model that processes inputs to generate outputs. During the learning process, the model updates weights to minimize the difference between predicted and real outputs. ANN can have multiple layers and complex structures, allowing them to solve intricate tasks.

#### Autoregressive integrated moving average (ARIMA)

ARIMA is a widely used method for time series prediction. It models a time series as a combination of autoregressive (AR) and moving average (MA) components to capture patterns, trends, and seasonal patterns in the data. ARIMA has three main components: Autoregressive (AR), Moving Average (MA), and Integrated (I). The Autoregressive (AR) component models the relationship between past and present values in a time series. The text describes the ARIMA model, which measures the dependence of the present value on its own past values^78^.

The model comprises three components: the Integrated (I) component, which differentiates the time series to achieve stationarity, resulting in a constant mean and variance over time; the Moving Average (MA) component, which accounts for the relationship between the current value and past forecast errors; and the Autoregressive (AR) component, which captures short-term irregularities in time series data. (p, d, q) represents the mathematical formula used for time series prediction. The value of p denotes the number of past observations considered for predicting the current value, while d indicates the number of times the time series data is different to make it stationary. Finally, q represents the number of past forecast errors used to predict the current value. ARIMA models use mathematical equations and statistical techniques to estimate the parameters of past forecast errors. These parameters are then used to predict the current value and project future values based on past data. Accurate parameter estimation, appropriate model selection, and diagnostic evaluations are essential for ensuring the effectiveness of ARIMA models.

#### Hybrid models

Two distinct hybrid deep learning models (CNN + LSTM and Attention + GRU) were employed to generate 10-year forward projections for the attributes contained within the traffic accident dataset utilised in this study. The two models were constructed utilising the sliding window approach and 5-fold cross-validation method to analyse time series data. The objective is to utilise historical data to enhance the precision of future fatality predictions.

Initially, the data underwent standardisation (for preprocessing integration^79,80^) through the implementation of MinMaxScaler. Subsequently, the data from the preceding four years (window size = 4) was utilised as a window, and model input-output pairs were created to predict the value for the subsequent year using the values within this window. The CNN + LSTM model incorporates a 1D convolution layer (Conv1D) to detect local patterns over time and a long-short term memory network (LSTM) to learn time dependencies. This structure endeavours to encapsulate both short-term fluctuations and long-term dependencies. The Attention + GRU model employs a GRU (Gated Recurrent Unit) layer augmented with an Attention layer to ascertain the significance of steps in sequential data. While GRU attains a comparable level of success to LSTM with a reduced parameter count, the incorporation of an attention mechanism enables the model to concentrate more intently on relevant time steps. It is noteworthy that both models utilise the Adam optimization algorithm and the MSE (mean squared error) loss function.

Five-fold cross-validation (KFold (n_splits = 5)) was applied in order to evaluate the performance of the models and obtain more reliable predictions. For each fold, the model was retrained, 10-year predictions were made using the last four data points, and the final predictions were obtained by taking the average of the folds. Model performance was evaluated using the mean absolute percentage error (MAPE) metric, comparing it to actual data. Furthermore, a sensitivity analysis was conducted to enhance the reliability of the models. In this analysis, the final input value was corrupted by ± 10%, and the effect of these small changes on the 10-year predictions was observed. Consequently, the model’s input sensitivity was evaluated, resulting in enhanced reliability. The results were supported by graphical representations, and the trends and predictions of different models were presented to the user in an intuitive manner.

This structure represents a sophisticated approach by virtue of the fact that it employs hybrid models that combine the time series processing capabilities and the advantages of deep learning, in contrast to traditional forecasting methods. The comparison of different architectures, such as CNN + LSTM and Attention + GRU, provides important information for model selection. Furthermore, techniques such as sliding window and cross-validation have been integrated as methods to support the model’s generalizability and accuracy.

#### Statistical metrics

Statistical metrics, such as R^2^, R, MAE, RMSEP, RMSE, MSE, and MAPE, can be used to assess the system’s performance^81^. The R^2^ value takes a value between 0 and 1^82^, while the R value can take a value between − 1 and 1. Other metrics are usually expressed in percentages. These metrics were employed to indicate the error level of the proposed approach^83^. In this phase, we used two statistical metrics, MAPE (Mean Absolute Percentage Error) and correlation coefficient (R), to determine the stability levels of all models used in future prediction. The equations for these metrics are given below, respectively.

3 $$\:MAPE=\:\frac{100\%}{n}\:x\:\sum\:_{i=1}^{n}\frac{\left|{\text{a}\text{c}\text{t}\text{u}\text{a}\text{l}\text{v}\text{a}\text{l}\text{u}\text{e}}_{\text{i}}-{\text{f}\text{o}\text{r}\text{e}\text{c}\text{a}\text{s}\text{t}\text{v}\text{a}\text{l}\text{u}\text{e}}_{\text{i}}\right|}{\left|{\text{a}\text{c}\text{t}\text{u}\text{a}\text{l}\text{v}\text{a}\text{l}\text{u}\text{e}}_{\text{i}}\right|}$$

4 $$\:R=\frac{\sum\:\left(X-\stackrel{-}{X}\right)(Y-\stackrel{-}{Y})}{\sqrt{\sum\:{(X-\stackrel{-}{X})}^{2}\sum\:{(Y-\stackrel{-}{Y})}^{2}}\:}$$

where,

$\:X$ and $\:Y$ are the values of two variables.

$\:\stackrel{-}{X}$ and $\:\stackrel{-}{Y}$ are the mean values of the variables.

MAPE (%) and R values obtained in this study are given in Table 2. As can be seen in the table, the prediction results of the 3 methods used in the study are very close to the actual results. Accordingly, 2-year (2021–2022) predictions are obtained by the models and MAPE and R values were calculated according to these predictions. Then, 10-year (2023–2032) forecasts were obtained without changing the models.

**Table 2:** Prediction values obtained for the methods applied between 2021–2022.

Parameters		2021						2022				
	Real	ANN	ARIMA	SSM	CNN + LSTM	Attention + GRU	Real	ANN	ARIMA	SSM	CNN + LSTM	Attention + GRU
Driver Fatalities	***11***	12	13	13	13	13	***13***	13	14	14	14	14
Passenger Fatalities	***13***	15	15	13	19	18	***19***	17	18	15	22	21
Pedestrian Fatalities	***18***	16	16	18	20	20	***19***	17	17	20	21	22
Injured Driver	***3721***	3481	3753	3730	4379	4336	***4177***	3636	4172	4146	4752	4705
Injured Passenger	***4227***	4293	4339	4391	5113	4976	***4608***	4696	4919	4971	5567	5441
Injured Pedestrian	***1178***	1150	1101	1160	1428	1459	***1328***	1259	1133	1288	1567	1615
Total Fatalities	***42***	40	40	43	51	52	***51***	41	45	48	56	56
Total Injuring	***8771***	8057	8732	8851	10,311	10,344	***9851***	8243	9460	9864	11,183	11,218
Total Accident	***9851***	4856	5021	5069	6180	6069	***5776***	5185	5518	5650	6789	6650

### Proposed approach

**Fig. 2:**
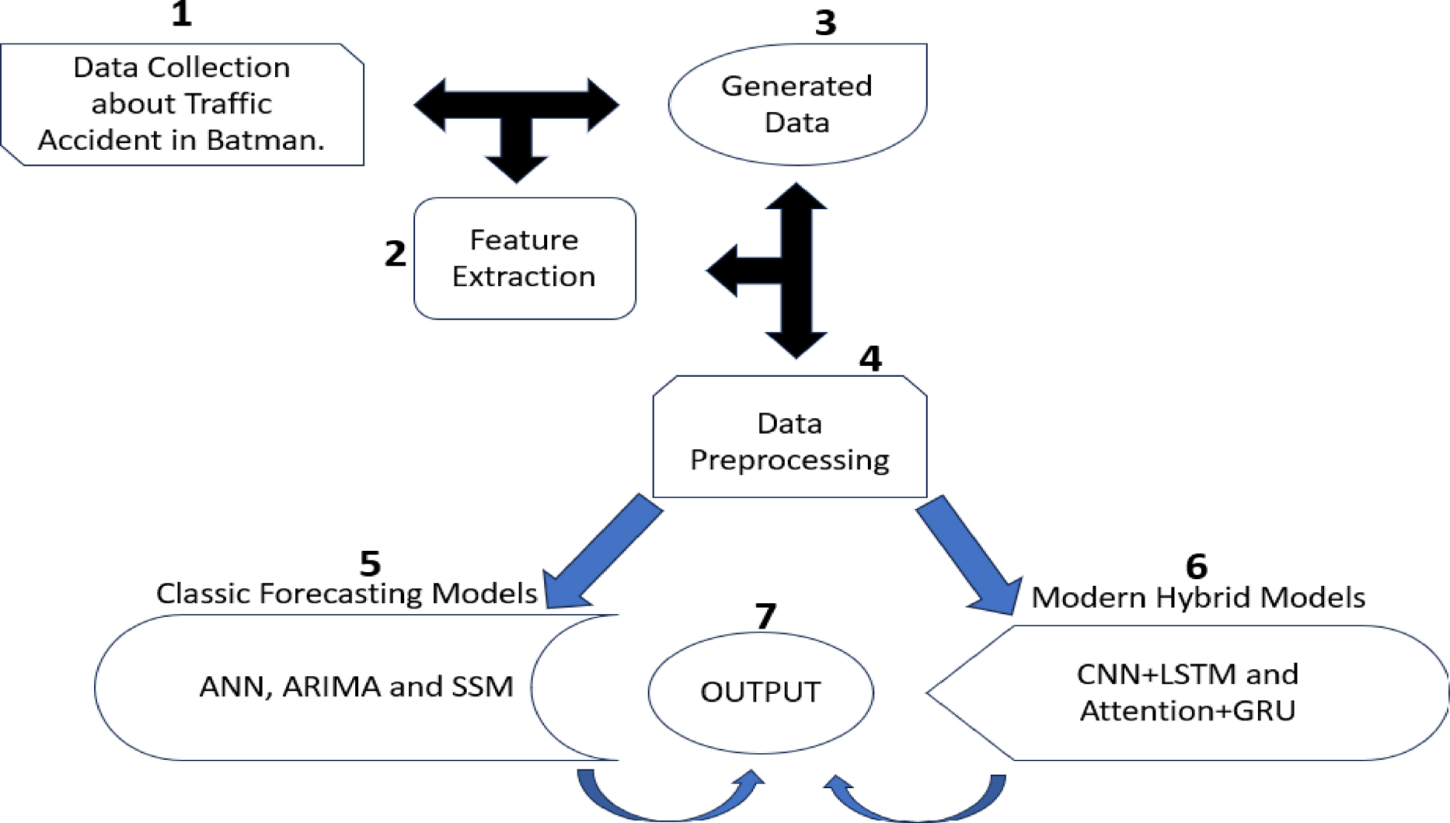
Work flow diagram of proposed approach.

The work flow chart of propesed approach is given at following step by steps and also is demonsrated in Fig. 2.

1. Data collection: The data set was obtained from the data of accidents occurring in Batman province between 2013 and 2022.
2. Feature extraction: Various data in the data set were collected and a new data set was obtained by combining these data. Nine different feature extraction processes were performed.
3. Generated data: Following data collection and feature extraction, the data set to be used in the study was created. This data set is shown in Table 1. The ratios of some features were found from this data set and examined together with the data set in terms of concepts such as mortality rate and injury rate.
4. Data preprocessing integration: After normalization was performed for each model in the data preprocessing steps and predictions were made, normalization was reversed to obtain the predicted values. In addition, the data set was adjusted to suit the models. In all models, the data set was allocated 80% for training and 20% for testing.
5. Classic forecasting models: Classic methods frequently used in time series forecasting were used in this study. These are ANN, ARIMA, and SSM models.
6. Modern hybrid models: The LSTM and CNN models, which are frequently used in prediction processes, were used as a hybrid model, and considering the size of the data set, the Attention + GRU model was also used as a second hybrid model for prediction.
7. Output: The expected forecast values for the future have been obtained at the output.

## Results and discussion

### ARIMA results

In the ARIMA method applied, the data in the data set were indexed under the year heading and pre-processed. Then, data were prepared for the model. Before training the prepared data, the most optimum p, q and d parameters were determined. These parameters were entered and training was performed. While obtaining the prediction, the first year of the prediction and how many steps the forecast will be made are added to the model. The prediction results obtained according to all these were plotted on the screen and printed numerically.

**Fig. 3:**
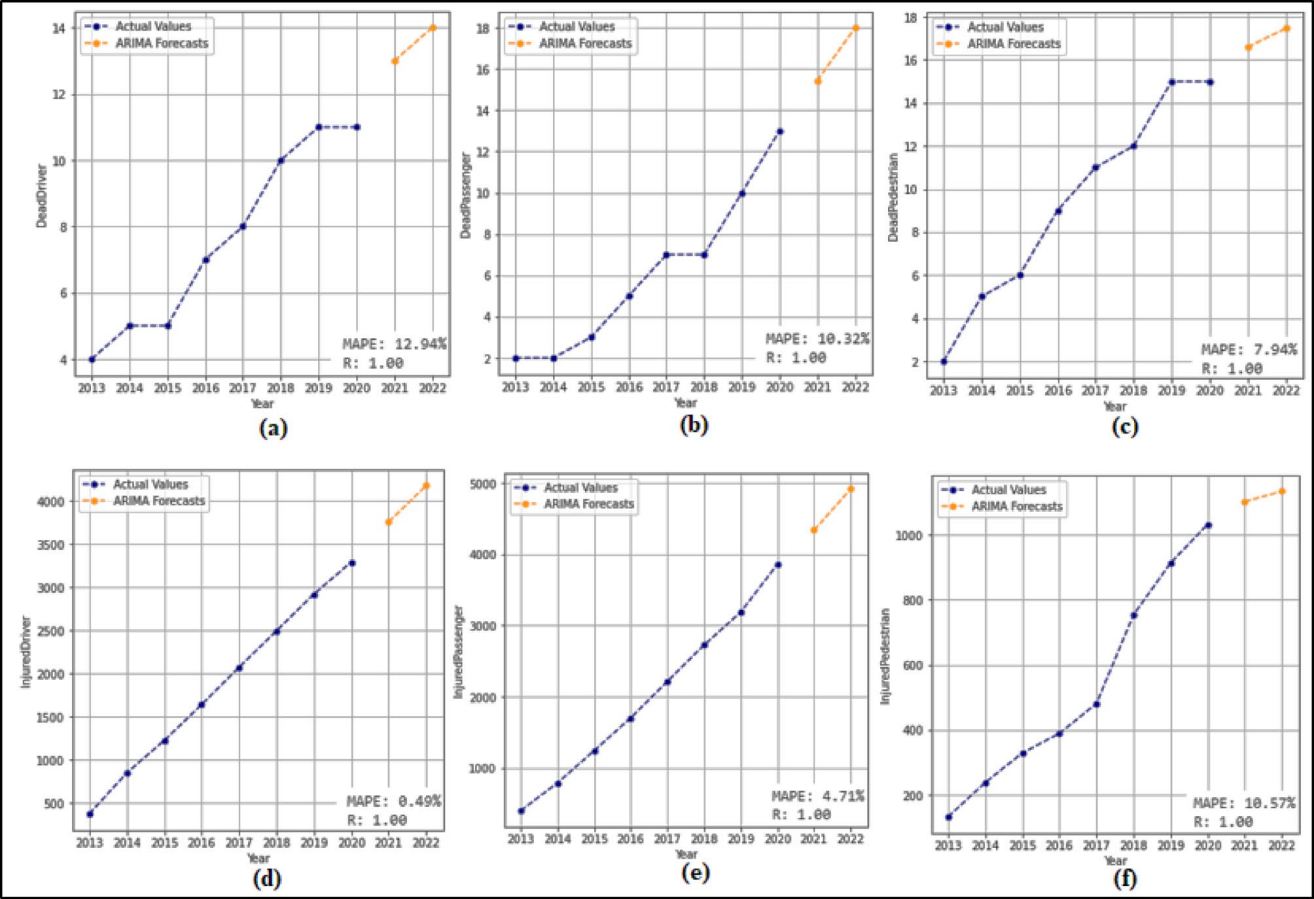
Prediction graphs obtained for the attributes (**a**) Driver Fatalities (**b**) Passenger Fatalities (**c**) Pedestrian Fatalities (**d**) Injured Driver (**e**) Injured Passenger (**f**) Injured Pedestrian.

**Fig. 4:**
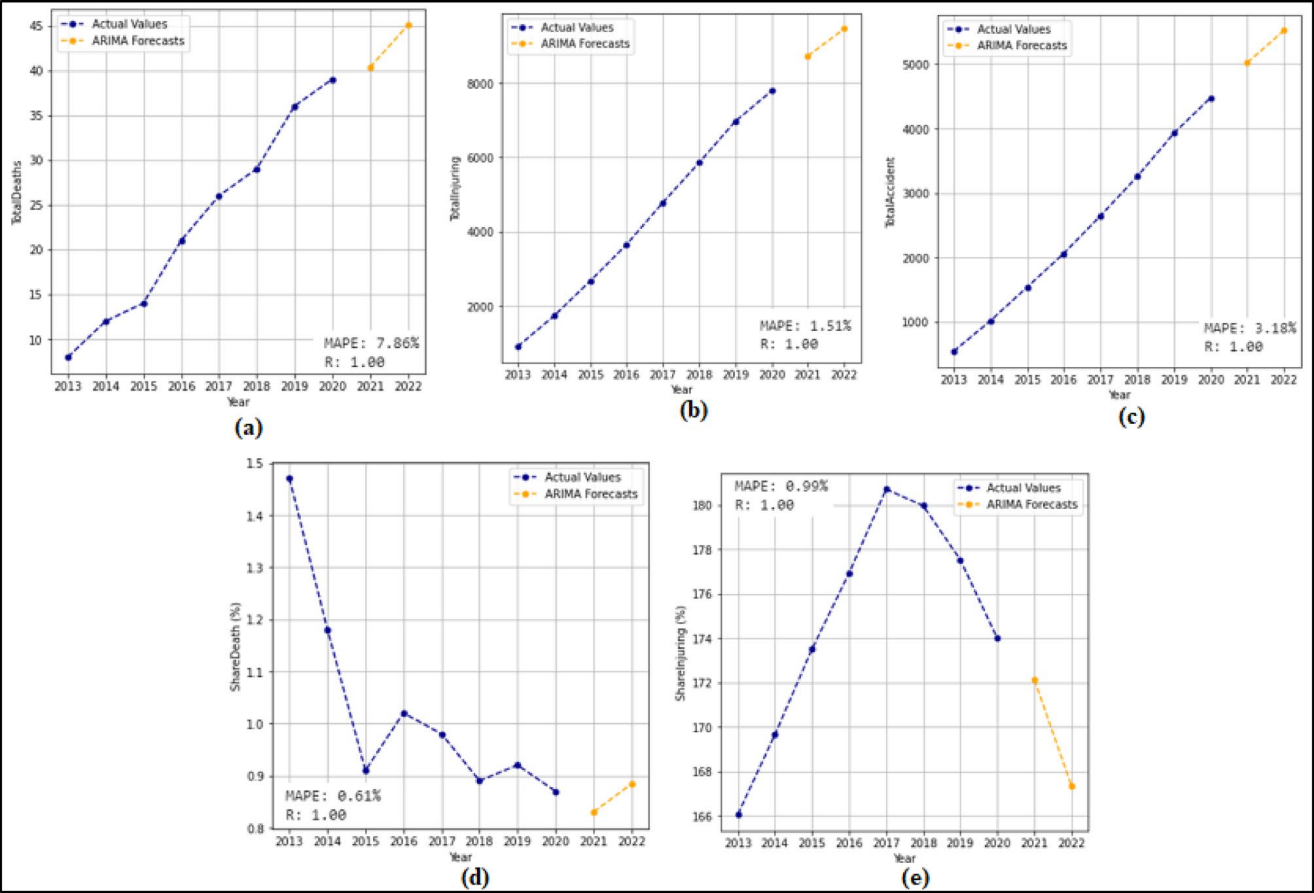
Prediction graphs obtained to forecast the attributes (**a**) Total Fatalities (**b**) Total Injuring (**c**) Total Accident (**d**) Share Death(%) (**e**) Share Injuring(%).

**Fig. 5:**
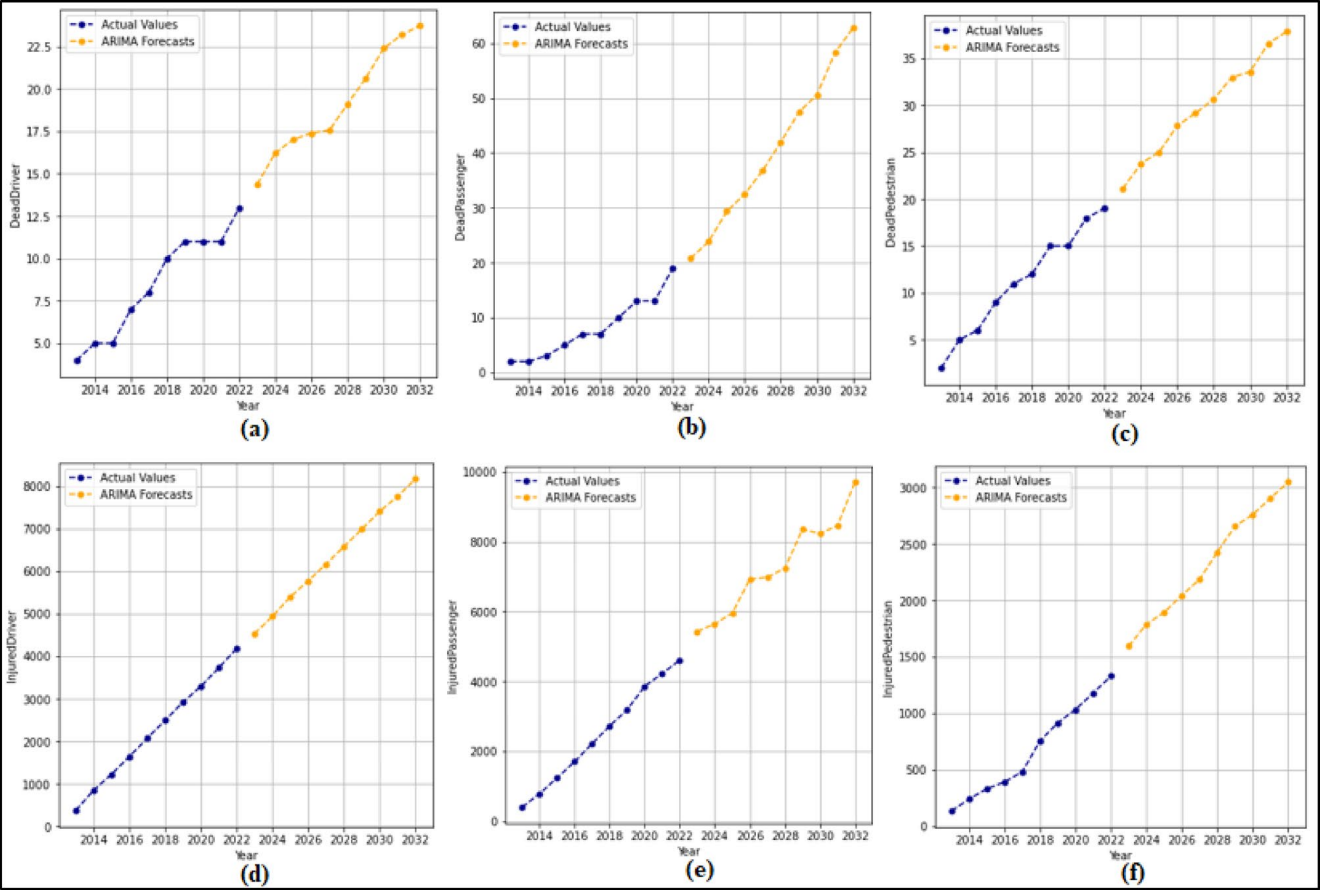
Prediction graphs generated using ARIMA to forecast the attributes (**a**) Driver Fatalities (**b**) Passenger Fatalities (**c**) Pedestrian Fatalities (**d**) Injured Driver (**e**) Injured Passenger (**f**) Injured Pedestrian.

**Fig. 6:**
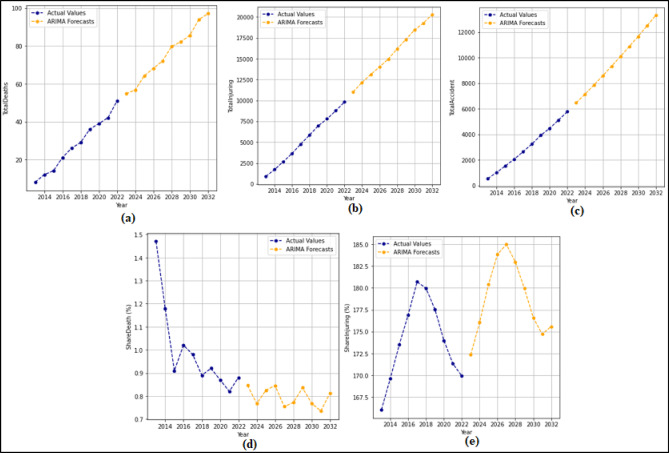
Prediction graphs generated using ARIMA to forecast the attributes (**a**) Total Fatalities (**b**) Total Injuring (**c**) Total Accident (**d**) Share Death(%) (**e**) Share Injuring(%).

In this section, 2-year (2021–2022) predictions are obtained by using the ARIMA model. MAPE and R values were calculated according to these predictions. Then, 10-year (2023–2032) predictions were obtained without changing the ARIMA model. For each feature in the dataset, 2-year predictions and 10-year predictions were obtained separately. The 2-year predictions obtained for each attribute and used to measure the stability of the model are shown in Fig. 3 and Fig. 4, and then the 10-year predictions are shown in Fig. 5 and Fig. 6.

.

Upon analyzing Fig. 3, it is evident that the ANN model predicts all attributes from 2021 to 2022, successfully. The MAPE (%) and R values have been determined based on these predictions. MAPE (%) and R values were found as (a) 12.94(%) and 1.00 (b) 10.32(%) and 1.00 (c) 7.94(%) and 1.00 (d) 0.49(%) and 1.00 (e) 4.71(%) and1.00 (f) 10.57(%) and 1.00. Figure 4 shows that the ARIMA model successfully predicts all attributes between 2021 and 2022. The MAPE (%) and R values determined based on these predictions are (a) 7.86(%) and 1.00 (b) 1.51(%) and 1.00 (c) 3.18(%) and 1.00 (d) 0.61(%) and 1.00 (e) 0.99 (%) and 1.00.

When examined in Fig. 5, it is observed that the ARIMA model predicts all attributes from 2023 to 2032. While making these predictions, the model used for the prediction between 2021 and 2022 was applied unchanged. When analyzed in Fig. 6, it is found that the ARIMA model predictions all attributes from 2023 to 2032. While making these predictions, the model used for the prediction between 2021 and 2022 was applied unchanged.

### SSM results

In the SSM method applied, the data in the data set were indexed under the year heading and pre-processed. Since the data show a cumulative increase during the model construction, add was used as a trend feature. In addition, since the data are annual data, the seasonal period is selected as 1. This feature is selected as 7 for weekly data and 12 for monthly data. After the model was established, the desired date ranges were separated as test and training, and then the prediction process was performed. The obtained prediction results were plotted on the screen and printed numerically.

**Fig. 7:**
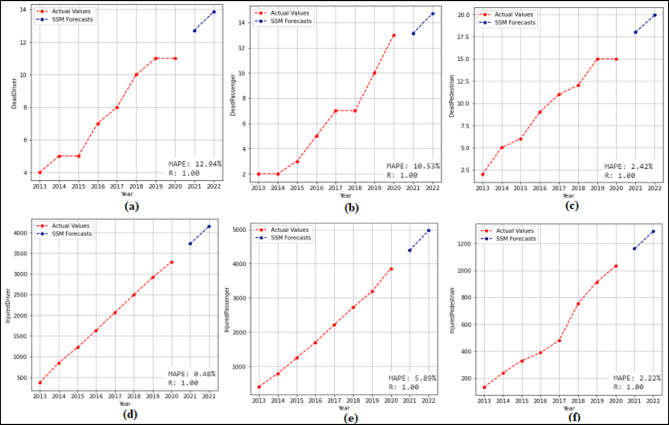
Prediction graphs obtained for the attributes (**a**) Driver Fatalities (**b**) Passenger Fatalities (**c**) Pedestrian Fatalities (**d**) Injured Driver (**e**) Injured Passenger (**f**) Injured Pedestrian.

**Fig. 8:**
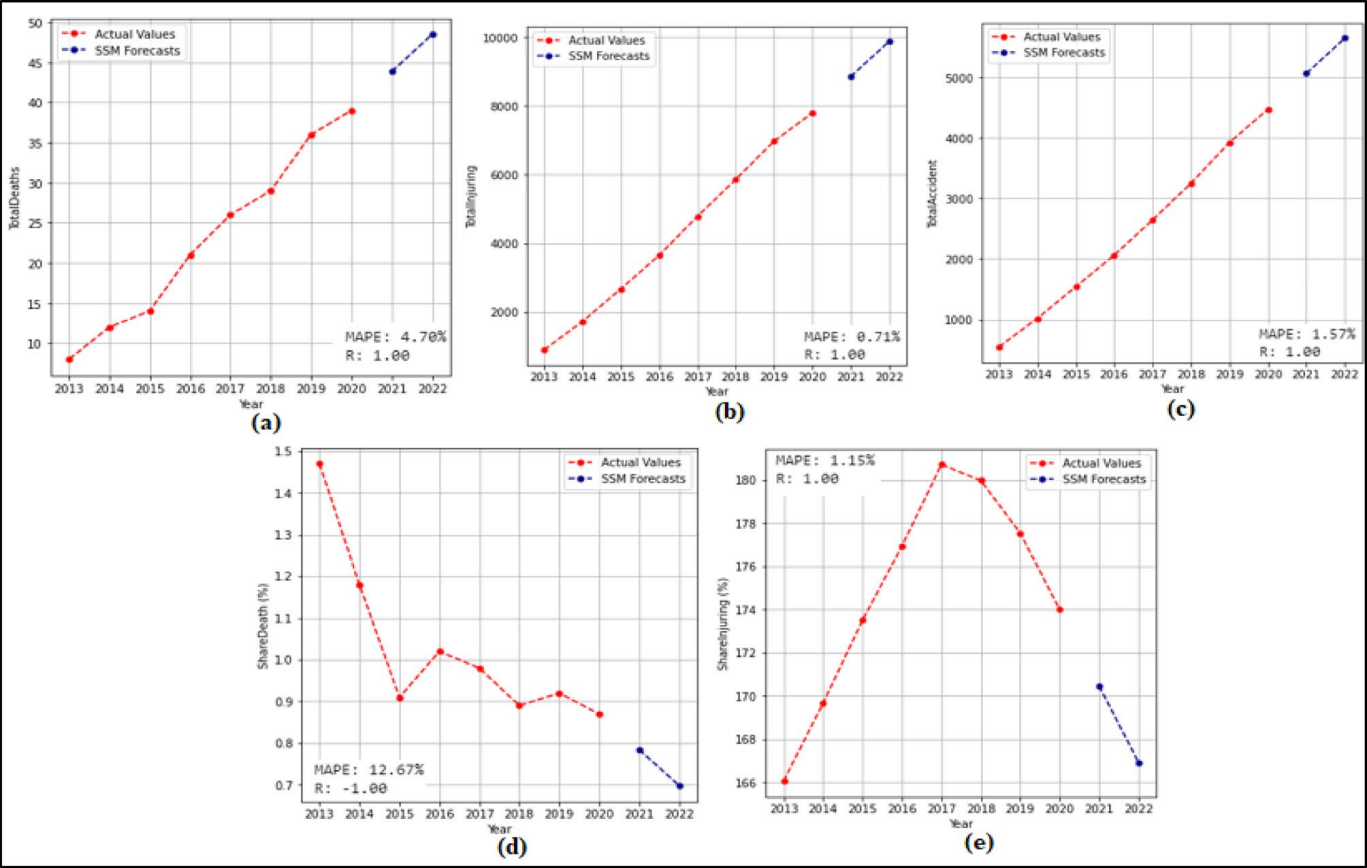
Prediction graphs obtained for the attributes (**a**) Total Fatalities (**b**) Total Injuring (**c**) Total Accident (**d**) Share Death(%) (e) Share Injuring(%).

**Fig. 9:**
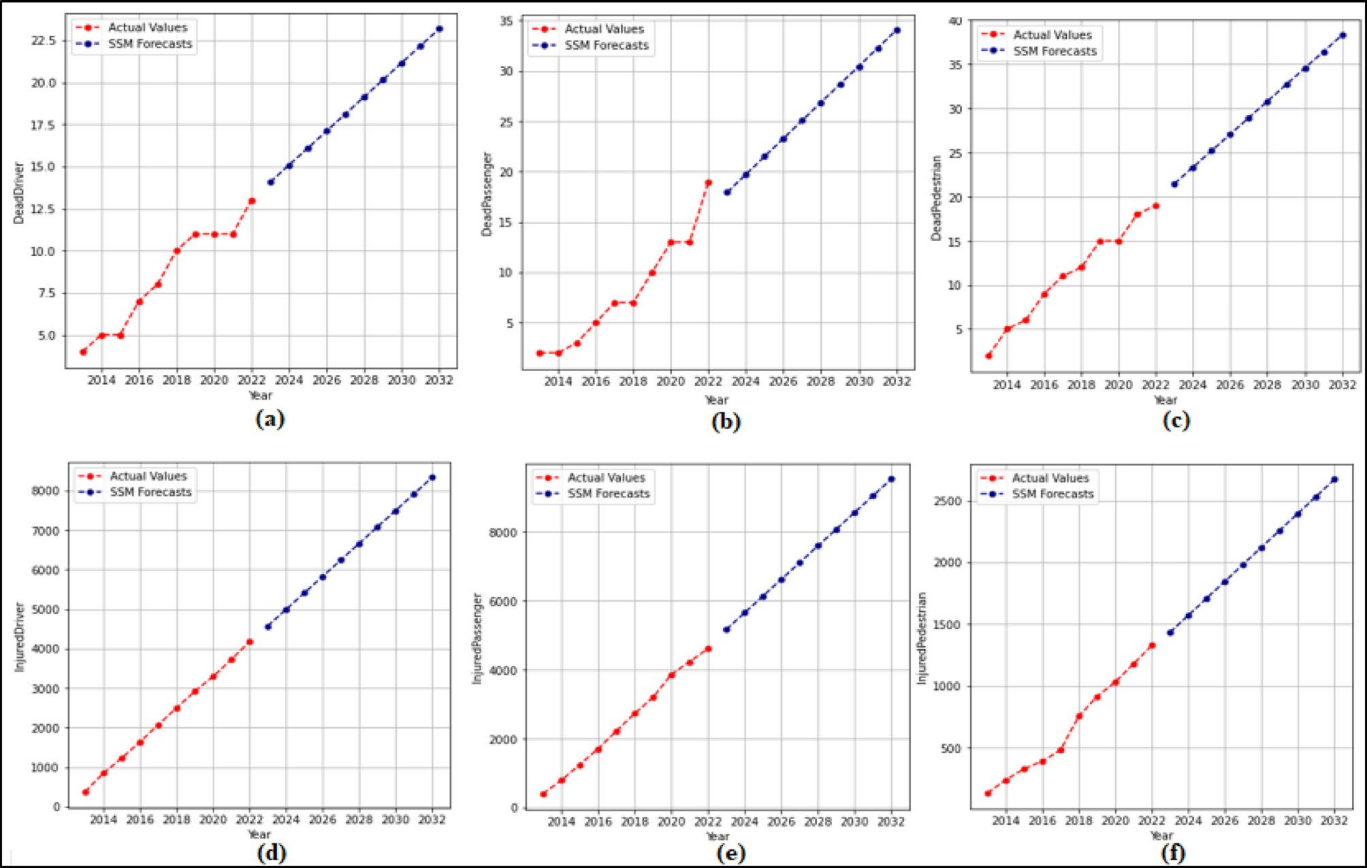
Prediction graphs generated using SSM for the attributes (**a**) Driver Fatalities (**b**) Passenger Fatalities (**c**) Pedestrian Fatalities (**d**) Injured Driver (**e**) Injured Passenger (**f**) Injured Pedestrian.

**Fig. 10:**
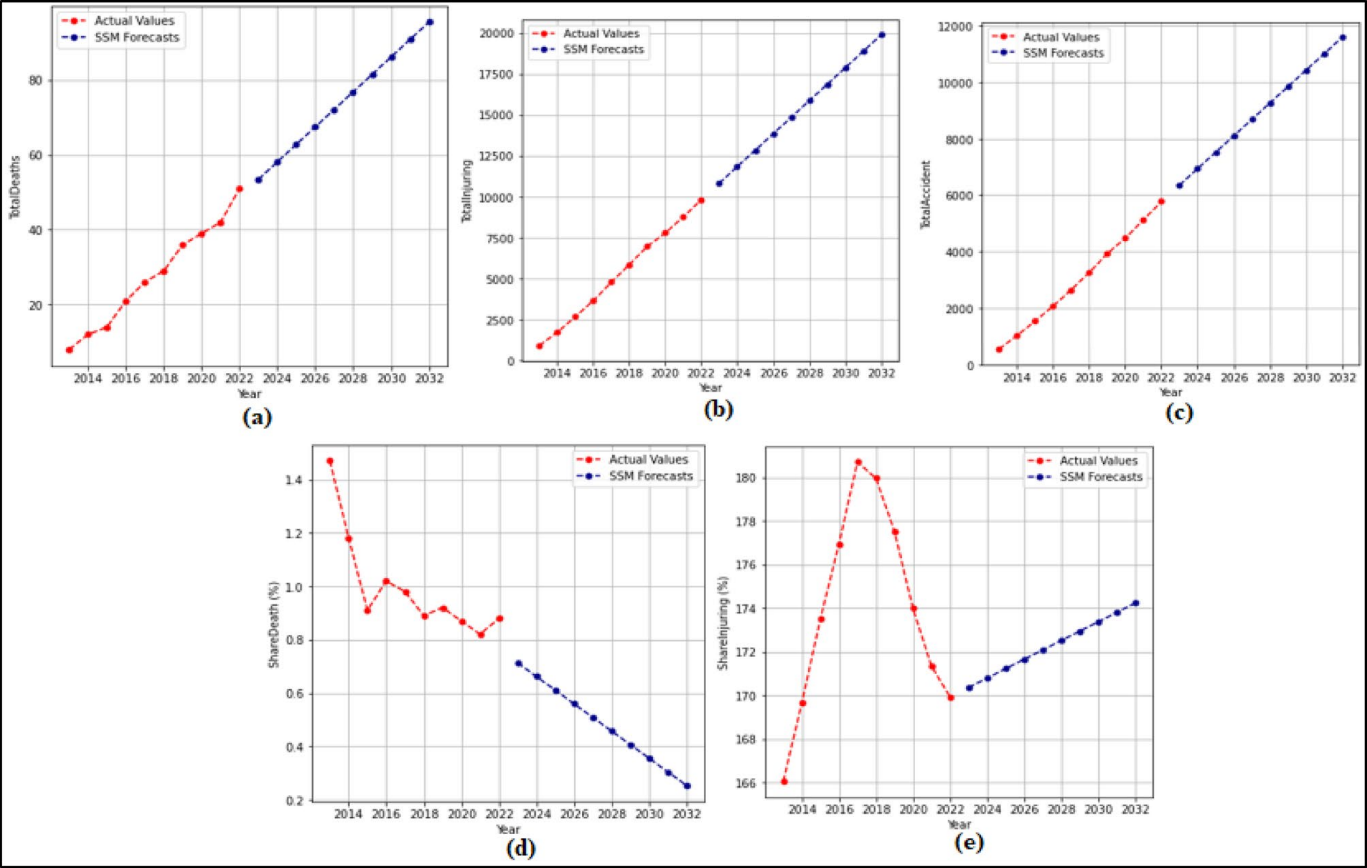
Prediction graphs generated using SSM for the attributes (**a**) Total Fatalities (**b**) Total Injuring (**c**) Total Accident (**d**) Share Death(%) (e) Share Injuring(%).

In this phase, 2-year (2021–2022) forecasts are received using the SSM model. According to these prediction, MAPE and R values were calculated. Then 10-year (2023–2032) predictions were received without changing the model. The 2-year and 10-year predictions were received separately for each attribute in the dataset. The 2-year prediction obtained for each attribute and used to measure the stability of the model are presented in Fig. 7 and Fig. 8, and then the 10-year predictions, where the possible future demand is predicted, are demonstrated in Fig. 9 and Fig. 10.

Figure 7 demonstrated that the SSM model successfully predicts all features except the TrHESProdGWh feature between 2021 and 2022. The MAPE (%) and R values calculated based on these prediction are (a) 12.94(%) and 1.00 (b) 10.53(%) and 1.00 (c) 2.42(%) and 1.00 (d) 0.48(%) and 1.00 (e) 5.89(%) and 1.00 (f) 2.22(%) and 1.00. Figure 8 presented that the SSM model predicts all features between 2021 and 2022. The MAPE (%) and R values calculated based on these prediction are (a) 4.70(%) and 1.00 (b) 0.71(%) and 1.00 (c) 1.57(%) and 1.00 (d) 12.67(%) and − 1.00 (e) 1.15(%) and 1.00.

As seen in Fig. 9, it is seen that the SSM model predictions all attributes between 2023 and 2032. While making these predictions the model used for the forecasting between 2021 and 2022 was reapplied without any changes. When analysed in Fig. 10, it is demonstrated that the SSM model predicts all attributes between 2023 and 2032. While making these predictions, the model used for the predictions between 2021 and 2022 was reapplied without any changes.

### ANN results

In the applied ANN method, firstly, the features are extracted from the data set in order and after the scaling (normalisation) process, the data set is divided into training and test parts. The data set was adjusted as 20% for testing and 80% for training. While building the model, 2 dense layers working with the ReLu activation function were added. The third dense layer was added for output and the activation function was chosen linearly. The model was then compiled and the results were obtained. The prediction results obtained by performing normalisation recycling in the results were plotted and printed numerically.

**Fig. 11:**
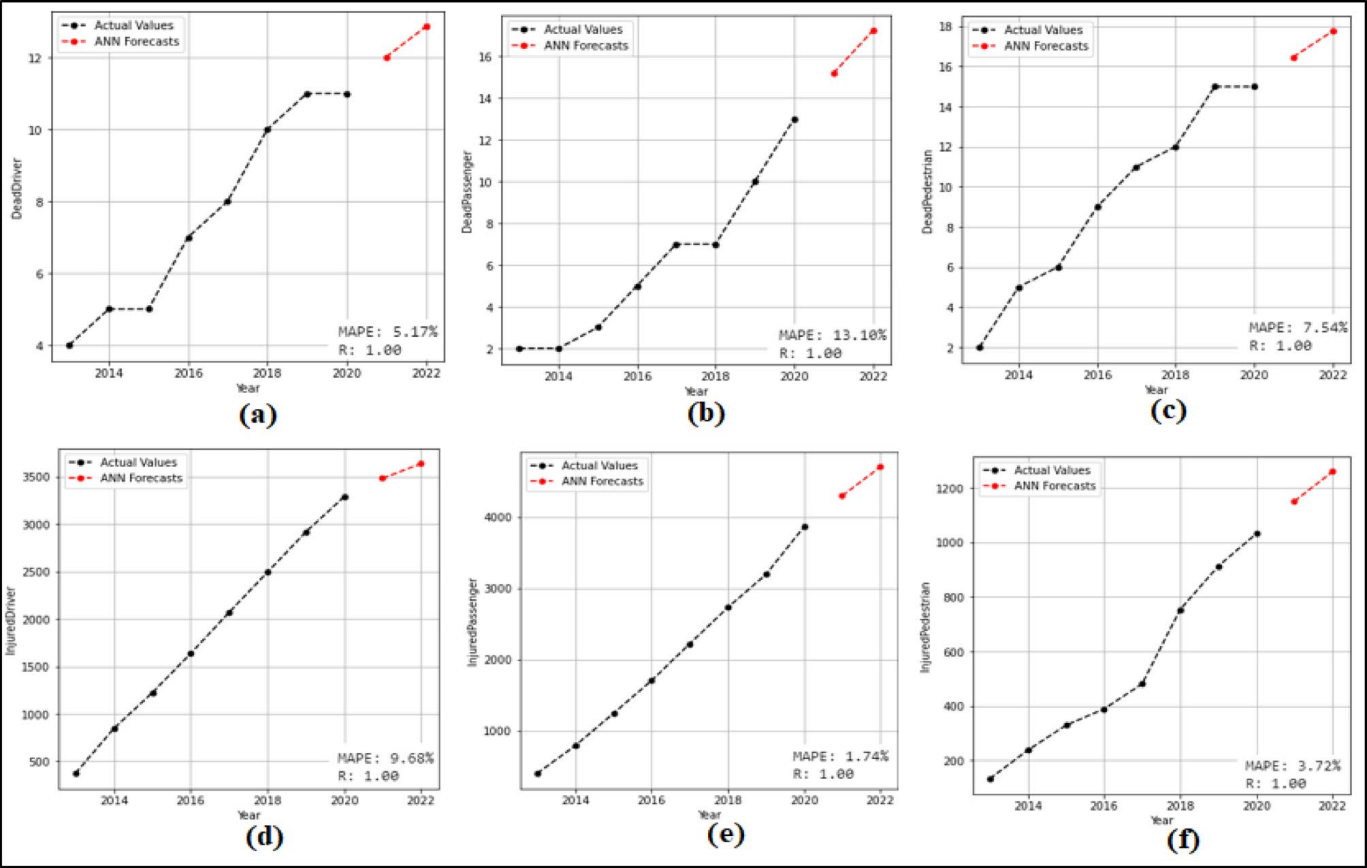
Predictions graphs obtained for the attributes (**a**) Driver Fatalities (**b**) Passenger Fatalities (**c**) Pedestrian Fatalities (**d**) Injured Driver (**e**) Injured Passenger (**f**) InjuredPedestrian.

**Fig. 12:**
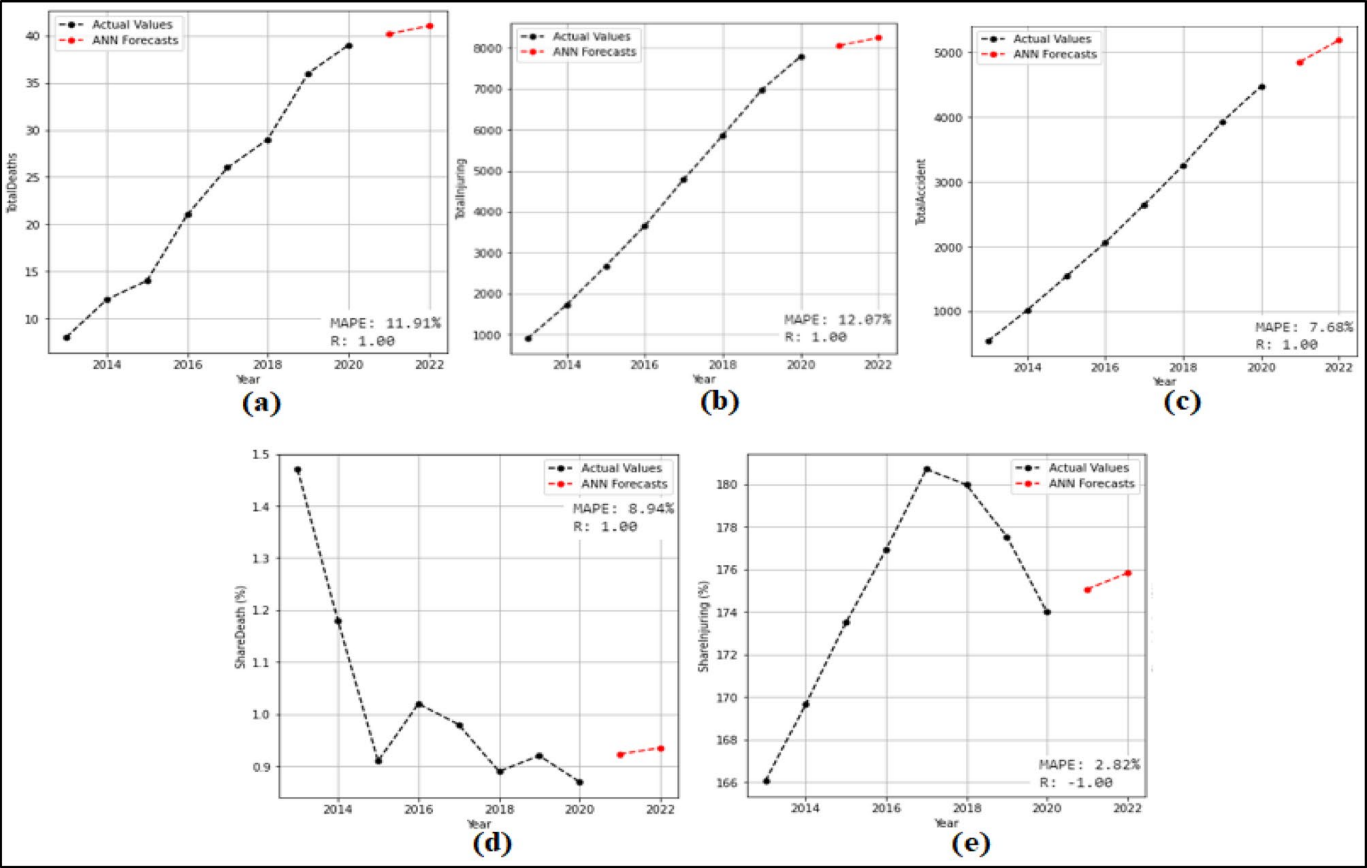
Predictions graphs obtained for the attributes (**a**) Total Fatalities (**b**) Total Injuring (**c**) Total Accident (**d**) Share Death(%) (**e**) Share Injuring (%).

**Fig. 13:**
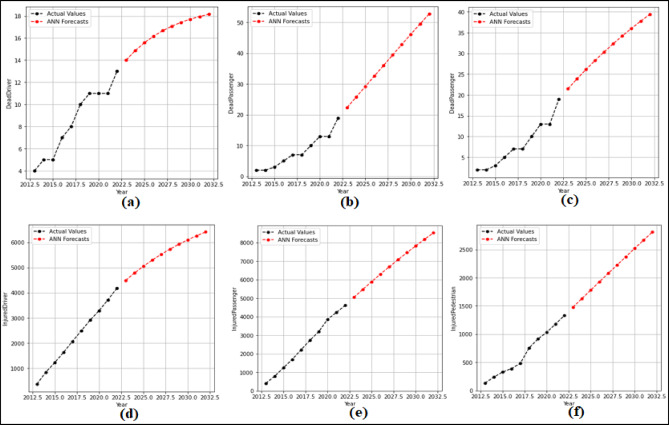
Predictions graphs generated using ANN to forecast the attributes (**a**) Driver Fatalities (**b**) Passenger Fatalities (**c**) Pedestrian Fatalities (**d**) Injured Driver (**e**) Injured Passenger (**f**) Injured Pedestrian.

**Fig. 14:**
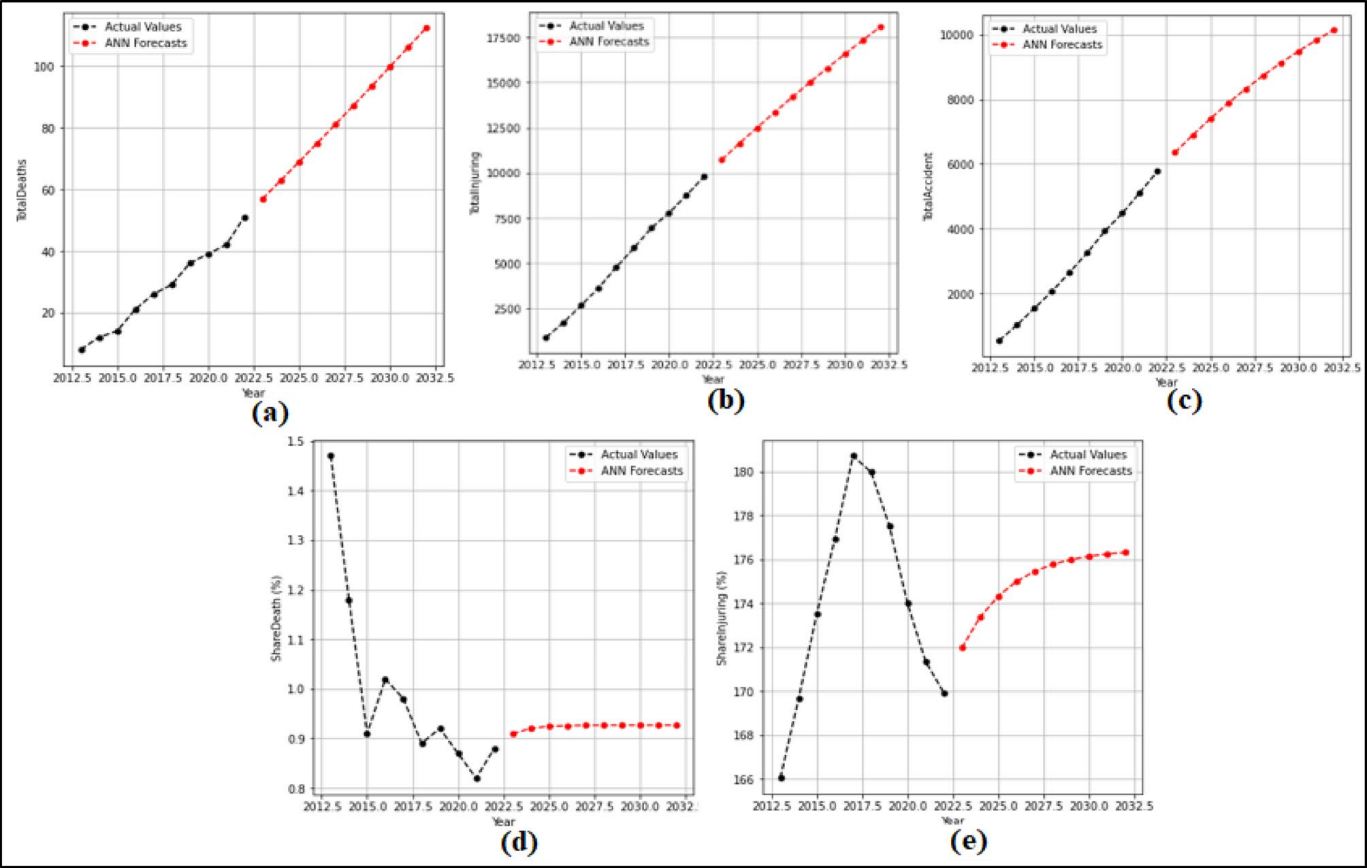
Predictions graphs generated using ANN to forecast the attributes (**a**) Total Fatalities (**b**) Total Injuring (**c**) Total Accident (**d**) Share Death(%) (**e**) Share Injuring(%).

At this stage, 2-year (2021–2022) predictions are obtained using the ANN model. According to these predictions MAPE and R values were calculated. Then 10-year (2023–2032) forecasts were obtained without changing the model. The 10-year forecasts were obtained separately for each attribute in the dataset. The 2-year predictions obtained for each attribute and used to measure the stability of the model are given in the Fig. 11 and Fig. 12, and then the 10-year forecasts, where the possible future demand is forecasted, are given in the Fig. 13 and Fig. 14.

When analyzed Fig. 11, it is evident that the ANN model predicts all attributes from 2021 to 2022, succesfully. The MAPE (%) and R values have been determined based on these predictions. MAPE (%) and R values were found as (a) 5.17(%) and 1.00 (b) 13.10(%) and 1.00 (c) 7.54(%) and 1.00 (d) 9.68(%) and 1.00 (e) 1.74(%) and 1.00 (f) 3.72(%) and 1.00. Figure 12 shown that the SSM model successfully predicts all features between 2021 and 2022. The MAPE (%) and R values calculated based on these predictions are (a) 11.91(%) and 1.00 (b) 12.07(%) and 1.00 (c) 7.68(%) and 1.00 (d) 8.94(%) and 1.00 (e) 2.82(%) and − 1.00. When analysed in Fig. 13, it is demonsrated that the ANN model forecasts all attributes between 2023 and 2032. While making these forecasts, the model used for the forecasts between 2021 and 2022 was reapplied without any changes. As seen in Fig. 14, it is seen that the ANN model forecasts all attributes between 2023 and 2032. While making these forecasts, the model used for the forecasts between 2021 and 2022 was reapplied without any changes.

### Hybrid models results

In the case of hybrid models used, features were initially extracted from the dataset in sequence. Following this, the dataset was subjected to scaling (normalization), and subsequently divided into training and testing sections. The dataset was divided into two parts: 20% was allocated for testing, and 80% for training. The model was then compiled and the results obtained. The prediction results obtained after normalisation reversal were displayed graphically and printed numerically.

**Fig. 15:**
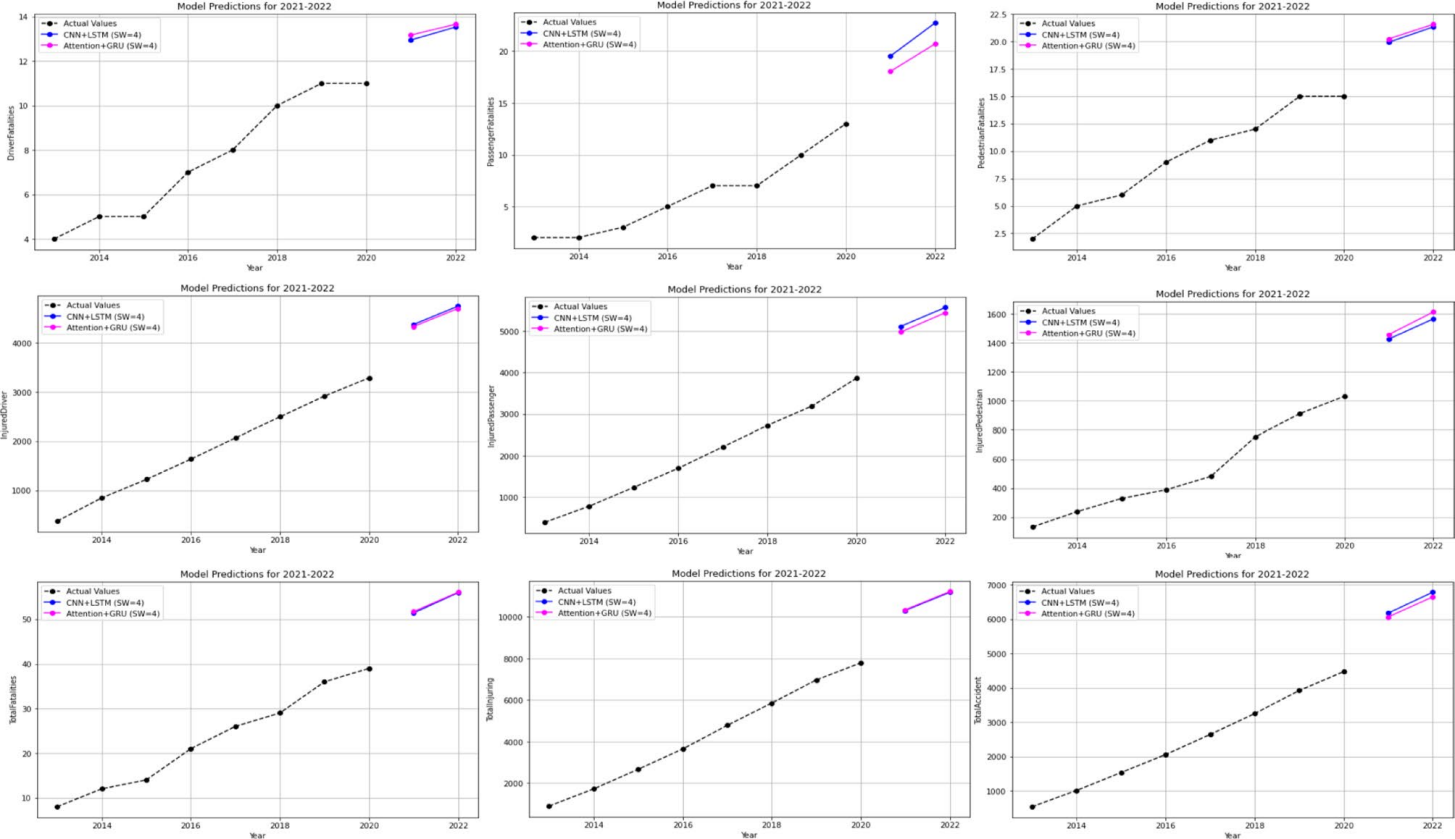
Predictions graphs generated using hybrid models to predict the all attributes.

**Fig. 16:**
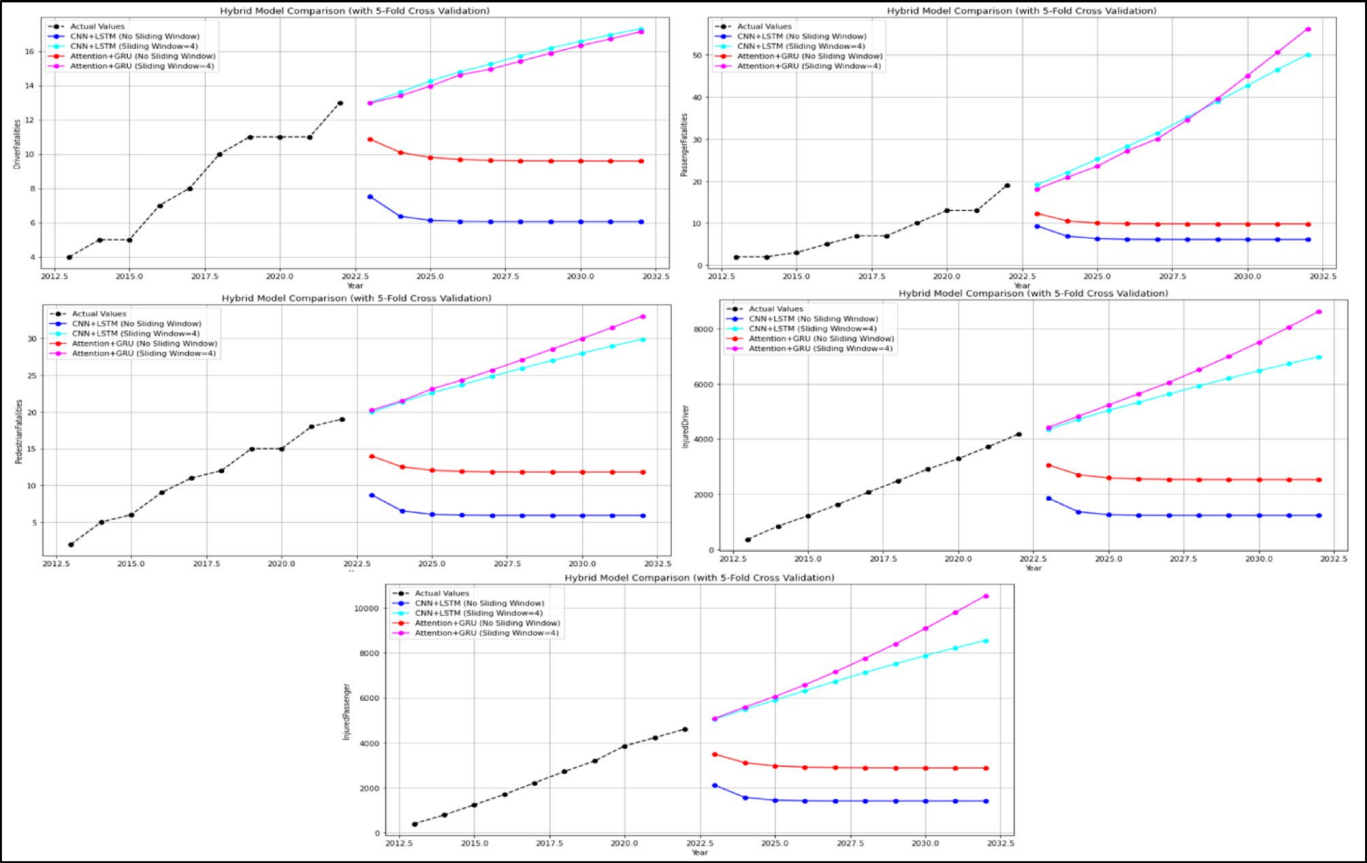
Forecasting graphs generated using Hybrid Models to forecast the first 5 attributes.

**Fig. 17:**
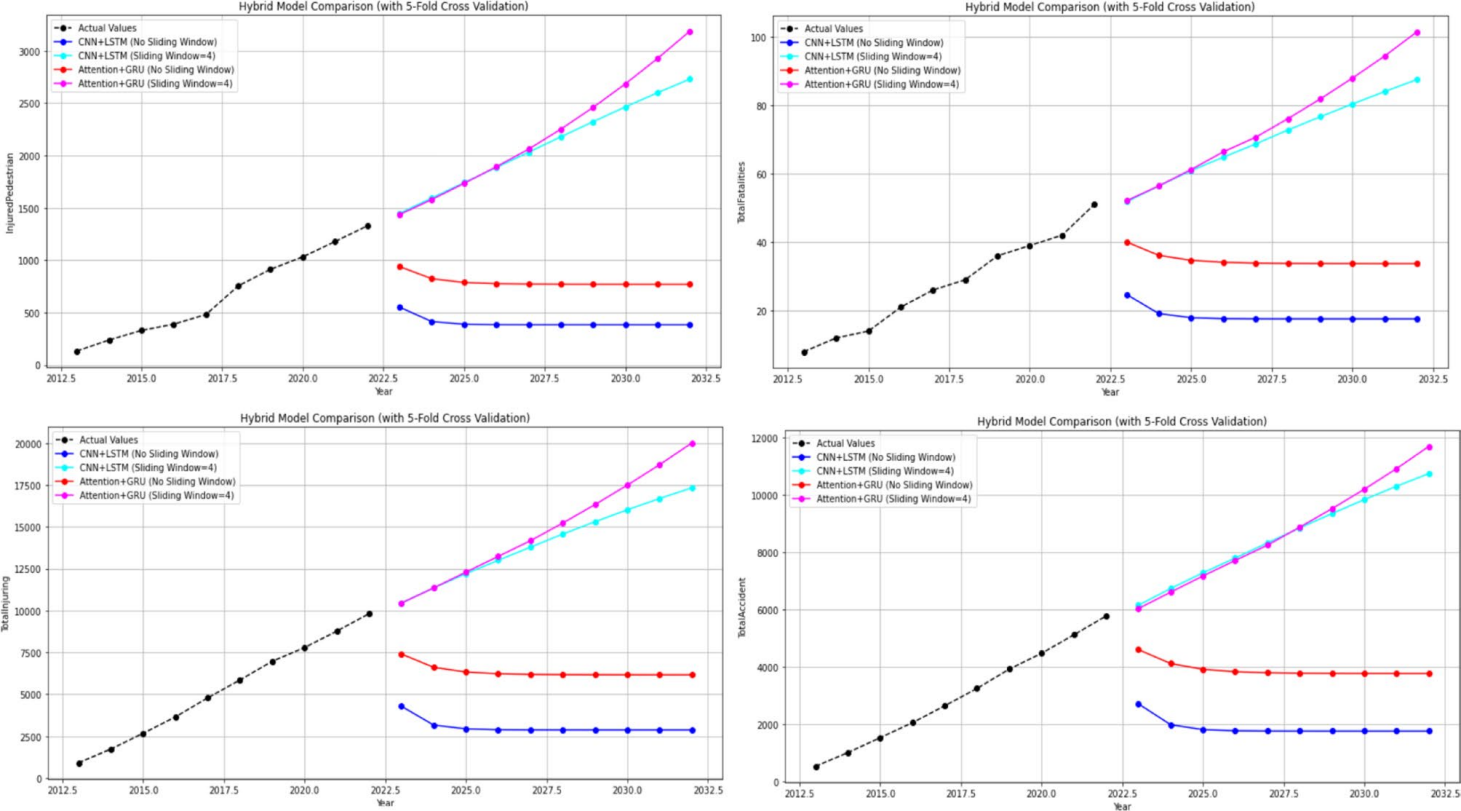
Forecasting graphs generated using Hybrid Models to forecast the last 4 attributes.

**Fig. 18:**
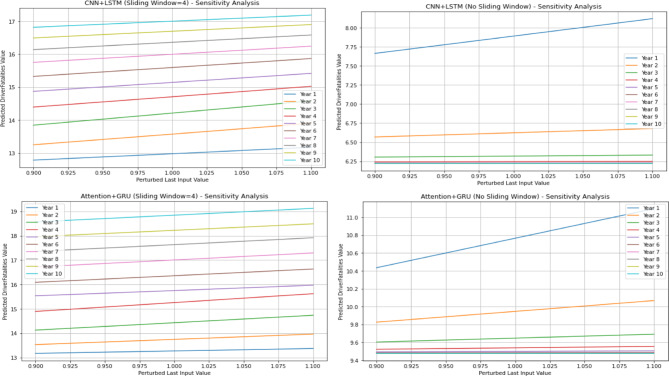
Sensitivity Analysis graphs of Hybrid Models.

At this stage, predictions for the 2-year period (2021–2022) were obtained using two different hybrid models (CNN + LSTM and Attention + GRU). The MAPE and R values were calculated based on these predictions. Subsequently, predictions for the 10-year period (2023–2032) were obtained without modifying the models. The 10-year forecasts were obtained separately for each feature in the dataset. The 2-year forecasts obtained for each feature and utilised to assess the model’s stability are presented in Figs. 15, while the 10-year forecasts predicting potential future demand are shown in Figs. 16 and 17. In addition, sensitivity analyses were performed to determine the sensitivity (with sliding window and without it) of these hybrid models established with 5-fold cross-validation, and these analyses were provided in Fig. 18 as an example. These analyses were performed for all attributes, and similar results were obtained in terms of sensitivity.

As illustrated in Fig. 15, the impact of the sliding window technique employed in hybrid models on model prediction outcomes is evident. It has been demonstrated that predictions made in the absence of the sliding window technique are both unsuccessful and unacceptable. Upon thorough examination of the mean absolute percentage error (MAPE %) and the R values obtained, it has been observed that the success achieved by hybrid models applied using 5-fold cross-validation in conjunction with the sliding window technique is at an acceptable level for all attributes. Upon examination of Figs. 16 and 17, it becomes evident that hybrid models possess the capability to predict future expected values. When these values are compared to those of other models, as demonstrated in Tables 3 and 4, it is evident that they make similar predictions. This finding serves to corroborate the efficacy of hybrid models in this regard. As illustrated in Fig. 18, the sensitivity analysis demonstrates that the predictions of the hybrid models exhibit a positive and linear response to an increase in the final input value. This situation demonstrates that changes in the models’ predictions are directly and predictably dependent on the input. In conclusion, this analysis has proven that the model is sensitive to input changes and that predictions are logically affected. The underlying reason for the inability to perform calculations such as causal variable analysis and feature importance ranking is that each feature is examined individually, and the most important feature is itself and is not dependent on another variable.

Summary of MAPE (%) and R values obtained for each model applied are presented in Table 3. According to the results of forecasting due to the ARIMA, SSMs, and ANN, the number of fatalities and injuries of Drivers, Passengers, and Pedestrians and total traffic accidents are presented in Table 4.

**Table 3:** Summary of MAPE (%) and R values for employed all models for.

Parameters	ARIMA	SSMs	ANN	CNN + LSTM	Attention + GRU
Driver Fatalities	12.94 and 1.00	12.94 and 1.00	5.17 and 1.00	10.90 and 1.00	12.39 and 1.00
Passenger Fatalities	10.32 and 1.00	10.53 and 1.00	13.10 and 1.00	34.93 and 1.00	23.90 and 1.00
Pedestrian Fatalities	7.94 and 1.00	2.42 and 1.00	7.54 and 1.00	11.51 and 1.00	12.97 and 1.00
Injured Driver	0.49 and 1.00	0.49 and 1.00	9.68 and 1.00	15.73 and 1.00	13.60 and 1.00
Injured Passenger	4.71 and 1.00	5.89 and 1.00	1.24 and 1.00	20.89 and 1.00	17.90 and 1.00
Injured Pedestrian	10.57 and 1.00	2.22 and 1.00	3.72 and 1.00	19.60 and 1.00	22.71 and 1.00
Total Fatalities	7.86 and 1.00	4.70 and 1.00	11.91 and 1.00	16.08 and 1.00	16.54 and 1.00
Total Injuring	1.51 and 1.00	0.71 and 1.00	12.07 and 1.00	15.75 and 1.00	16.11 and 1.00
Total Accident	3.18 and 1.00	1.57 and 1.00	7.68 and 1.00	19.12 and 1.00	16.85 and 1.00

**Table 4:** Data of forecasting results.

Year	Models	Fatalities			Injured			Total		
		Driver	Passenger	Pedestrian	Driver	Passenger	Pedestrian	Fatalities	Injuring	Accident
2022	REAL DATA	13	19	19	4177	4608	1328	51	9815	5776
2023	ANN	14	22	21	4493	5047	1479	56	10,732	6354
	ARIMA	14	21	21	4526	5429	1593	54	11,040	6476
	SSM	14	18	21	4572	5162	1434	53	10,830	6356
	CNN + LSTM	13	19	20	4347	5054	1448	52	10,453	6150
	Attention + GRU	13	18	20	4421	5081	1436	52	10,445	6034
2024	ANN	14	25	23	4785	5476	1629	62	11,628	6895
	ARIMA	16	24	24	4933	5646	1786	56	12,155	7140
	SSM	15	19	23	4990	5647	1571	58	11,837	6937
	CNN + LSTM	14	22	21	4710	5488	1594	56	11,352	6731
	Attention + GRU	13	21	21	4823	5588	1579	56	11,351	6606
2025	ANN	15	29	26	5053	5893	1779	68	12,502	7401
	ARIMA	17	29	25	5390	5957	1893	64	13,115	7865
	SSM	16	21	25	5408	6132	1709	62	12,844	7518
	CNN + LSTM	14	25	23	5036	5898	1741	61	12,201	7278
	Attention + GRU	14	24	23	5231	6056	1733	61	12,293	7168
2026	ANN	16	32	28	5299	6300	1929	74	13,356	7875
	ARIMA	17	33	28	5768	6926	2043	68	14,012	8575
	SSM	17	23	27	5826	6617	1846	67	13,851	8099
	CNN + LSTM	15	28	24	5326	6318	1885	64	12,999	7793
	Attention + GRU	15	27	24	5636	6578	1893	66	13,233	7709
2027	ANN	16	36	30	5525	6696	2077	80	14,190	8317
	ARIMA	18	37	29	6161	6982	2184	72	14,963	9340
	SSM	18	25	28	6244	7102	1984	72	14,858	8680
	CNN + LSTM	15	31	25	5625	6729	2031	69	13,768	8320
	Attention + GRU	15	30	26	6042	7152	2060	71	14,170	8241
2028	ANN	17	39	32	5733	7082	2225	87	15,003	8732
	ARIMA	19	42	31	6570	7239	2426	79	16,155	10,080
	SSM	19	26	30	6661	7587	2121	76	15,865	9261
	CNN + LSTM	16	35	26	5920	7127	2179	73	14,566	8841
	Attention + GRU	15	35	27	6507	7757	2252	76	15,226	8868
2029	ANN	17	42	34	5925	7458	2372	93	15,798	9119
	ARIMA	21	47	33	6980	8348	2655	82	17,287	10,878
	SSM	20	28	32	7079	8072	2259	81	16,871	9841
	CNN + LSTM	16	39	27	6199	7511	2323	77	15,306	9342
	Attention + GRU	16	40	29	6992	8397	2459	82	16,330	9512
2030	ANN	17	46	36	6101	7824	2519	99	16,573	9482
	ARIMA	22	51	34	7400	8237	2759	85	18,427	11,655
	SSM	21	30	34	7497	8558	2396	86	17,878	10,422
	CNN + LSTM	17	43	28	6470	7879	2464	80	16,016	9827
	Attention + GRU	16	45	30	7504	9083	2684	88	17,489	10,188
2031	ANN	17	49	37	6262	8181	2664	105	17,330	9821
	ARIMA	23	58	37	7754	8452	2904	93	19,248	12,487
	SSM	22	32	36	7914	9042	2534	90	18,885	11,003
	CNN + LSTM	17	47	29	6731	8226	2600	84	16,692	10,293
	Attention + GRU	17	51	31	8048	9805	2928	94	18,712	10,908
2032	ANN	18	52	39	6411	8528	2807	112	18,066	10,138
	ARIMA	24	63	38	8159	9697	3047	97	20,252	13,298
	SSM	23	34	38	8333	9528	2671	95	19,892	11,584
	CNN + LSTM	18	50	30	6978	8552	2729	87	17,329	10,734
	Attention + GRU	18	56	33	8626	10,546	3188	101	20,007	11,679
Increase Rate Between 2032 Forecasting Data and 2022 Real Data	ANN	0,38	1,74	1,05	0,53	0,85	1,11	1,20	0,84	0,76
	ARIMA	0,84	2,32	1,00	0,95	1,10	1,29	0,90	1,06	1,30
	SSM	0,77	0,79	1,00	0,99	1,07	1,01	0,86	1,03	1,01
	CNN + LSTM	0,38	1,63	0,58	0,67	0,86	1,06	0,71	0,77	0,86
	Attention + GRU	0,38	1,95	0,74	1,07	1,29	1,40	0,98	1,04	1,02

As seen from Table 3, the models have higher accuracy and the highest ones are expressed in bold. However, the better accuracy due to the parameters were determined with SSMs compared to the others. The higher accuracy obtained with ARIMA and ANN based models are determined with only two parameters. This showed that SSMs is the most appropriate machine learning models for forecasting the future trajectory of traffic accidents on a majority basis and their results within the studied parameters. As analyzed data in Table 4, it clearly shows that there is a dramatic increase in traffic accident occurrence and respectively death and injured people. Although the models give different increase rates between 2032 forecasting data and 2022 real data, the rate is almost twofold. In addition, although the success of the hybrid models used is lower than the classical methods, it is observed that the forecasts obtained for future forecasts are very close to the other model forecasts. This reveals the success of hybrid methods.

In Table 5, some current studies in the literature employing different learning techniques to forecast traffic accident rates are given. Looking at the table, it can be seen that two approached the best result with a very low MAPE error rate compared to t-similar studies.

**Table 5:** The comparative table of similar studies in the literature.

Reference	Road Accident Forecasting Method	Contenxt	Metric	Performances
^61^	ANN BN	Forecasting of Injuries and Mortality as a result of traffics accident in Switzerland	MAPE	ANN = 30,0% BNN = 51,8%
^63^	ARIMA	Forecasting of Fatality as a result of traffic accidents in India	MAPE	ARIMA = 5,67%
^65^	SARIMA ERNN	To evaluate road traffic accidents in China	MAPE	SARIMA = 5,04% ERNN = 4,83%
^67^	SARIMA SSM	To predict monthly traffic accident cases in Malaysia	MAPE	SARIMA = 3,267% SSM = 2,845%
^36^	MVNB SVM SSM-SVM	To predict traffic accidents in the state of Tennessee, USA	MAPE	MVNB = 14,147% SVM = 11,840% SSM-SVM = 3,522%
^69^	SSM	To predict of traffic accident fatalities for the next 10 years in India	MAPE	5,84%
Proposed study	ANN ARIMA SSM Hybrid Models (CNN + LSTM and Attention + GRU)	Forecasting of Injuries and Fatalities as a result of traffic accidents for next 10 year in Batman, Turkey	MAPE	ANN = 2,20% ARIMA = 1,90% SSM = 1,86% CNN + LSTM = 10,90% Attention + GRU = 12,97%

## Conclusion

This study utilized traffic accident data from the years 2013 to 2022 to forecast the expected trajectory of accidents in Batman province through 2032 using ARIMA, ANN, SSM and Hybrid methods. Although these models are well-established, our study provides an important contribution by applying them to a region that has not previously been the focus of such detailed predictive modelling. Batman, located in southeastern Turkey, is undergoing rapid urbanization and changes in traffic density, infrastructure, and vehicle usage patterns. These conditions create unique traffic dynamics that justify focused modelling and analysis.

Based on the applied models, the following conclusions were drawn:

- The models produced varying results, emphasizing the importance of comparative modelling approaches.
- Among all, the SSM model yielded the highest accuracy across most parameters.
- The historical trend (2013–2022) already shows a clear and concerning increase in traffic accidents and casualties.
- Forecasting analysis for 2023–2032 indicates a dramatic escalation in the number of accidents, deaths, and injuries if no interventions are implemented.
- To provide acceptable forecasts with modern hybrid methods.

This region-specific prediction not only fills a critical gap in the national traffic safety literature but also offers actionable insights for local authorities. The results can guide the development of tailored countermeasures including infrastructure improvement, targeted traffic enforcement, and public safety campaigns. These steps are essential for mitigating human losses and should be prioritized by local and national decision-makers.

We recommend that similar predictive studies be conducted in other understudied regions in Turkey, using both traditional and advanced hybrid modelling approaches. This will enable a more comprehensive national traffic safety strategy supported by regional data and risk profiling.

In particular, pedestrian and passenger safety should be prioritized based on the observed rise in injuries among these vulnerable road users. Urban areas with high accident density, especially near schools and intersections, should receive focused traffic enforcement and improved road signage.

Moreover, our findings should be used as a decision-support tool for regional traffic authorities. By integrating these forecasts into strategic planning, municipalities can optimize emergency response allocation, schedule infrastructure upgrades more effectively, and evaluate the expected impact of planned interventions under realistic growth conditions.

## Data Availability

Data will be available from the corresponding author on reasonable request.
